# Germline Defects Caused by *Smed-boule* RNA-Interference Reveal That Egg Capsule Deposition Occurs Independently of Fertilization, Ovulation, Mating, or the Presence of Gametes in Planarian Flatworms

**DOI:** 10.1371/journal.pgen.1006030

**Published:** 2016-05-05

**Authors:** Jessica Kathryne Steiner, Junichi Tasaki, Labib Rouhana

**Affiliations:** Department of Biological Sciences, Wright State University, Dayton, Ohio, United States of America; Stowers Institute for Medical Research, UNITED STATES

## Abstract

Few animals are known to lay eggs in the absence of ovulation or copulation, as it is presumably energetically wasteful and subjected to negative selection. Characterization of *Smed-boule*, a member of the DAZ family of germline RNA-binding proteins, revealed that egg capsule (or capsule) production and deposition occurs independently of the presence of gametes in the planarian flatworm *Schmidtea mediterranea*. Reduction of *Smed-boule* expression by RNA-interference (RNAi) causes ablation of spermatogonial stem cells and the inability of ovarian germline stem cells to undergo oogenesis. Although animals subjected to *Smed-boule* RNAi lose their gametes and become sterile, they continue to lay egg capsules. Production of sterile capsules is even observed in virgin *Smed-boule(RNAi)* and control planarians maintained in complete isolation, demonstrating that egg production in *S*. *mediterranea* occurs independently of ovulation, fertilization, or mating. Evidence suggests that this is a conserved feature amongst Platyhelminthes, and therefore relevant to the pathology and dissemination of parasitic flatworms. These findings demonstrate that *Smed-boule* functions at different stages during male and female germline stem cell development, and also demonstrate that egg capsule production by planarian flatworms occurs independently of signals produced by mating or ova.

## Author Summary

Our work shows that production and deposition of egg capsules by planarian flatworms does not require fertilization, mating, ovulation, or even the existence of gametes. We also uncovered evidence for the existence of gender-specific germline stem cells in *Schmidtea mediterranea*, a hermaphroditic species of flatworm that develops germ cells post-embryonically. These findings surfaced from the characterization of *Smed-boule*, a member of the *Deleted in AZoospermia* gene family of RNA-binding proteins required for germline development in a broad range of animals. These findings lead to a better appreciation of the evolutionary diversity in approaches to oviparity. Additionally, discovering that egg capsule production occurs independently of germline or mating activities may carry a potential applied aspect with regards to regulating the dissemination and pathology of parasitic flatworms (such as blood flukes and tapeworms), if conserved in these organisms.

## Introduction

The characterization of developmental processes involved in sexual reproduction has important implications towards reproductive medicine, stockbreeding, farming, and for controlling the dissemination of infectious disease. Evolutionarily conserved molecular processes involved in metazoan germline development have been identified through decades of research using model organisms. For example, post-transcriptional regulation of gene expression by conserved germline-specific RNA-binding proteins is one of the conserved molecular processes that ensure development of gametes [1–3]. On the other hand, there is great diversity in the processes that occur during and after fertilization, many of which are the outcome of speciation events [4,5].

Planarian flatworms belong to the phylum Platyhelminthes, and are well known for their extraordinary regenerative abilities, which are founded in the availability of a pluripotent stem cell population throughout their life [6–9]. The evolutionary history of these organisms has yielded extreme divergence of reproductive strategies, both between and within populations of different planarian species. For example, there are planarians that rely exclusively or temporally on asexual reproduction, which involves transverse fission and stem cell driven regeneration [7,10]. There are also populations of planarians that reproduce predominantly through parthenogenesis (Pongratz et al., 2003). However, the default reproductive strategy of turbellarians is believed to be hermaphroditic sexual reproduction [11], more specifically for planarians, through cross-fertilization and oviparity [12]. By contrast, some parasitic flatworms (i.e. schistosomes or blood flukes) have complex life cycles that involve dioecious and asexual reproductive phases during transitions between vertebrate and invertebrate hosts, respectively [13]. Since the complex life cycle of schistosomes complicates husbandry and experimentation in laboratory settings, researchers have begun to use planarian flatworms as a model to dissect the molecular mechanisms behind the extensive lifespan and reproduction of their parasitic cousins [14]. One aspect of particular interest is the continuous production of thousands of eggs that both facilitate dissemination and sustain the pathology of schistosomes by populating organs of their host [13,14].

Planarian flatworms have become useful models for the study of metazoan germline development [12,15]. In general, the specification of germline stem cells can occur through mechanisms that involve: 1) inherited material deposited in the cytoplasm of the maturing oocyte (preformation); or 2) embryonic stem cell differentiation in response to inductive cell-to-cell interactions (epigenesis) [16,17]. Inductive determination occurs in mice and is also observed in planarians, both initially and during regeneration of fragments that lack germ cells, and it occurs through differentiation of pluripotent somatic stem cells called neoblasts [18]. In the planarian species *Schmidtea mediterranea*, germline stem cells are first detected as dorsolateral clusters in the area where testes develop [18]. In other planarian species, such as *Dugesia ryukyuensis*, germline stem cells are first detected in the area of the ovaries [19,20]. Upon feeding and growth, planarians that reproduce sexually develop a hermaphroditic reproductive system and their gonads begin continuous production of gametes [12,19]. Germline stem cells in the ovary enter oogenesis and produce oocytes of approximately 40 μm in diameter that exit the ovary and are fertilized by sperm deposited in the tuba [12,15]. Even-though oocytes are large in comparison to other planarian cells (e.g. neoblasts are ~8 μm diameter) these do not hold the nutrients necessary for embryonic development, as is normally observed in eggs of insects, amphibians or fish (to name a few). Instead, planarian yolk glands (vitellaria in other flatworms) produce separate cells that provide material required for egg capsule shell formation and nurturing embryonic development [21,22].

The development of planarian germline stem cells depends on conserved post-transcriptional regulators such as Nanos and Bic-C [15,18]. Boule is an RNA-binding protein encoded by the basal member of the *Deleted in AZoospermia (DAZ)* gene family, which is required for germ cell development in species ranging from sea anemone to humans [23,24]. How *DAZ* family homologs contribute to germline development in planarians remains unknown. In this study, we characterize a Boule homolog in the planarian *Schmidtea mediterranea* and demonstrate that it functions at different stages during male and female germline development. Functional analyses by RNA-interference (RNAi) revealed that *Smed-boule* is required for development and maintenance of spermatogonial stem cells, but disposable for the existence of their oogonial counterparts, uncovering the presence of sex-specific germline stem cells in planarian hermaphrodites. Long-term analysis of *Smed-boule* knockdowns revealed that egg capsule deposition in planarians is not triggered by gametogenesis, ovulation, oocyte activation, fertilization, or mating. These results demonstrate that egg capsule formation occurs regardless of signals from sexual activity or germ cell activity in *S*. *mediterranea*. These findings also provide a unique opportunity to identify internal mechanisms that influence capsule production in Platyhelminthes, which is central in the dissemination and pathology of parasitic members of this phylum.

## Results

### *Smed-boule* is required for male and female germline development

**Fig 1:**
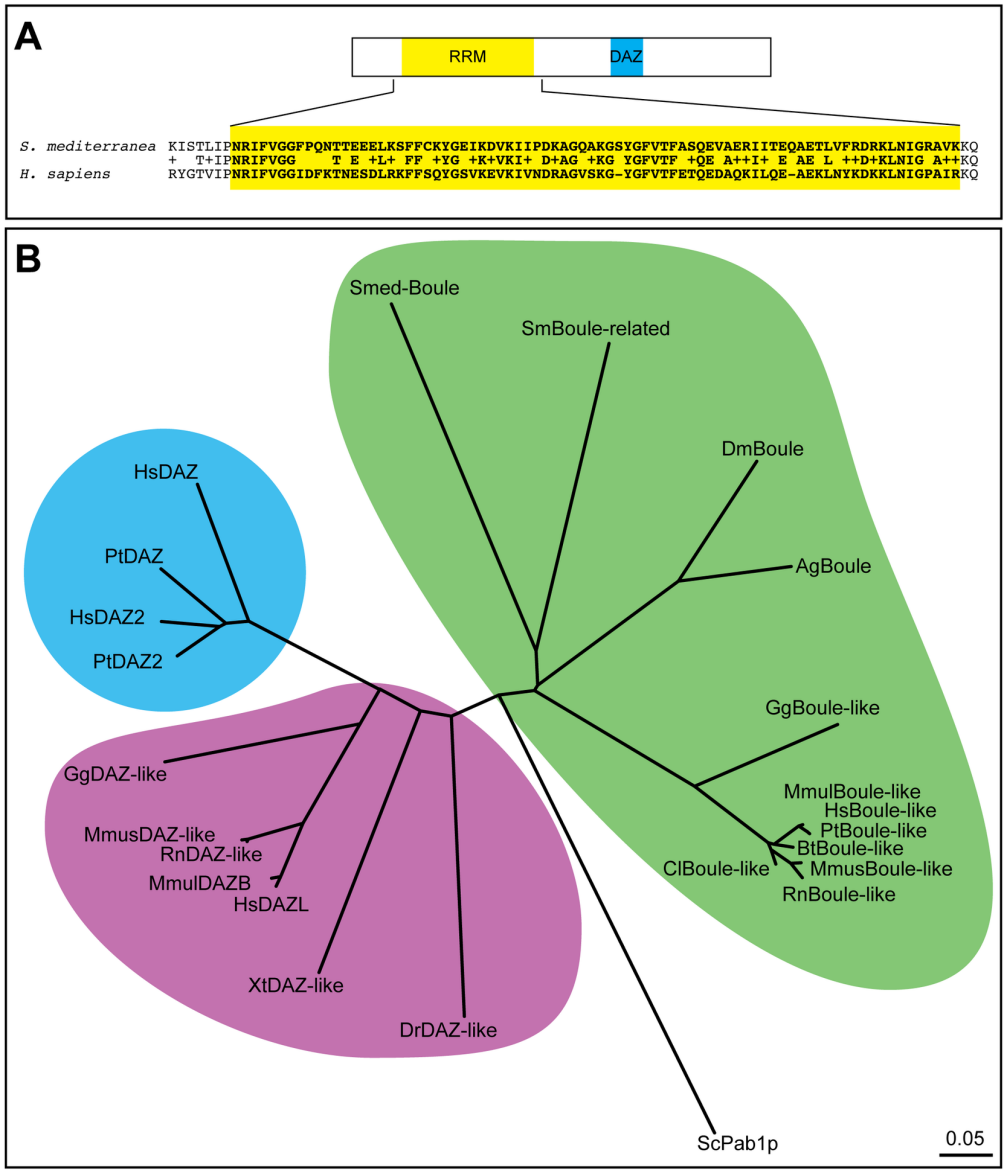
*Smed-boule* encodes for a member of the germline-specific Boule-Like subfamily of proteins. **(A)** Illustration of human Boule-Like (BOLL) protein architecture, which includes a RNA-recognition motif (RRM; yellow) and a DAZ domain (blue). The amino acid sequence conservation within the RRM of *Schmidtea mediterranea* and *Homo sapiens* homologs is shown. **(B)** Neighbor-joining phylogenetic tree depicting the closer association of Smed-Boule predicted amino acid sequence with members of the Boule-Like (green) subfamily of DAZ proteins, than with members of the DAZ (blue) and DAZ-Like (magenta) subfamilies. Phylogenetic analysis was performed using Clustal Omega with default parameters [44] and sequences obtained from NCBI accession NP_932074.1, XP_001169371.2, XP_001086915.2, XP_005640556.1, NP_001095585.1, NP_083543.2, XP_006245003.1, XP_004942650.1, NP_001261614.1, XP_315505.3, NP_001005785.1, XP_001138045.3, XP_002803072.1, NP_034151.3, NP_001102884.1, NP_989549.1, NP_571599.1, NP_989079.1, NP_004072.3, XP_003319020.2, CCD81039, NP_011092, NP_001177740.1. Abbreviations used for species names included *Anopheles gambiae* (Ag), *Bos taurus* (Bt), *Canis lupus familiaris* (Cl), *Danio rerio* (Dr), *Drosophila melanogaster* (Dm), *Gallus gallus* (Gg), *Homo sapiens* (Hs), *Macaca mulatta* (Mmul), *Mus musculus* (Mmus), *Pan troglodytes* (Pt), *Rattus norvegicus* (Rn), *Saccharomyces cerevisiae* (Sc), *Schmidtea mediterranea* (Smed), *Schistosoma mansoni* (Sm), and *Xenopus tropicalis* (Xt). Scale bar represents 0.05 substitutions per amino acid position.

**Fig 2:**
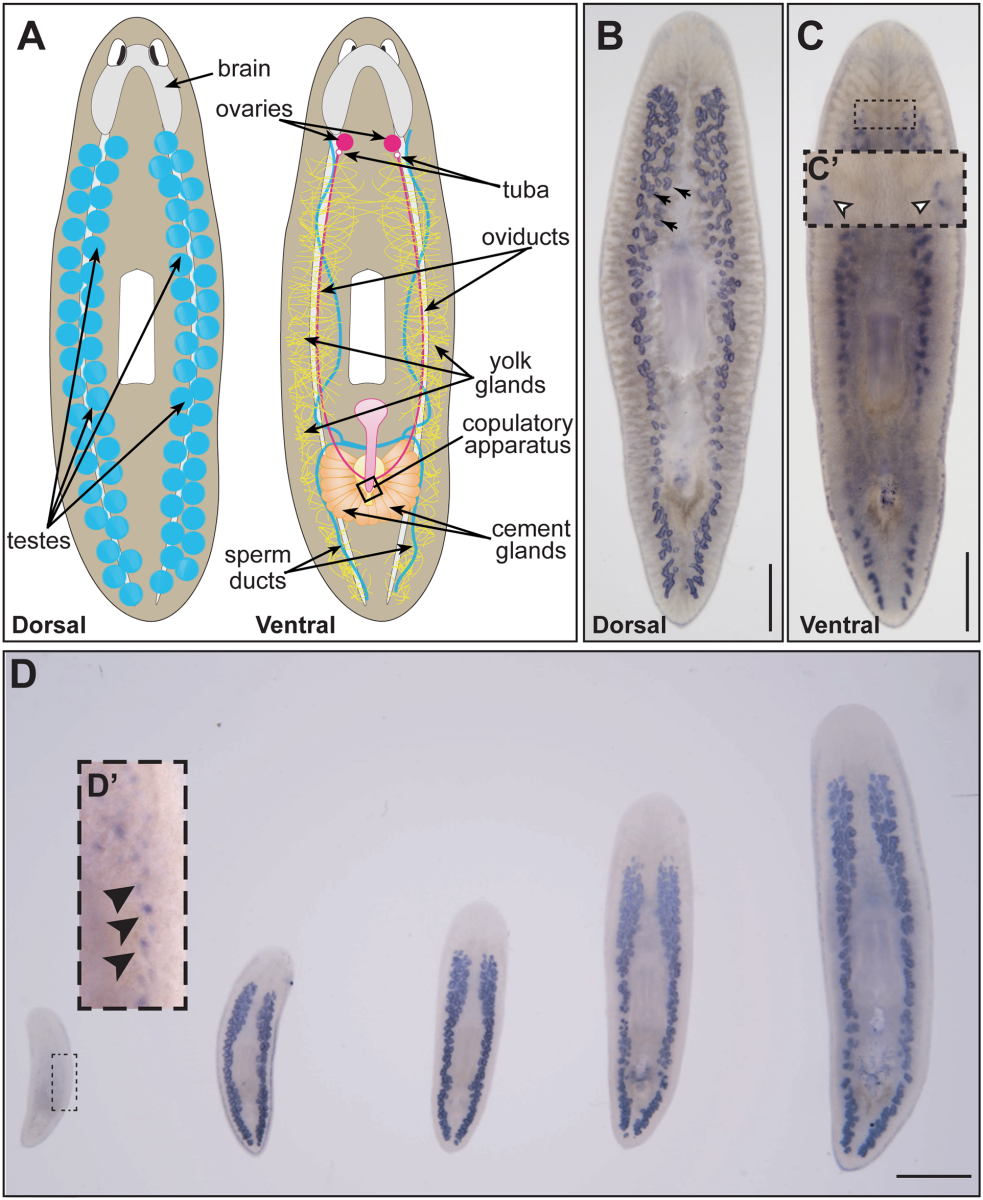
*Smed-boule* expression is restricted to the planarian germline. **(A)** Simplified depiction of the reproductive anatomy of planarian hermaphrodites. Reproductive organs located dorsally (top) and ventrally (bottom) are illustrated separately. **(B-D)** *Smed-boule* expression is detected in testes and ovaries by whole mount *in situ* hybridization in dorsal **(B and D)** and ventral **(C)** views of sexually mature *S*. *mediterranea*, respectively. Arrows in **(B)** indicate individual testis lobes within testes regions found dorsolaterally in the animal. Magnified view of signal from ovaries (open arrowheads) is shown in **(C’)** inset. *Smed-boule* expression is also detected in germline stem cells (black arrowheads) of 1 week-old hatchlings in the dorso-lateral region where testes develop **(D’)**, as well as continuously in maturing animals **(D).** Scale bars = 1 mm.

**Fig 3:**
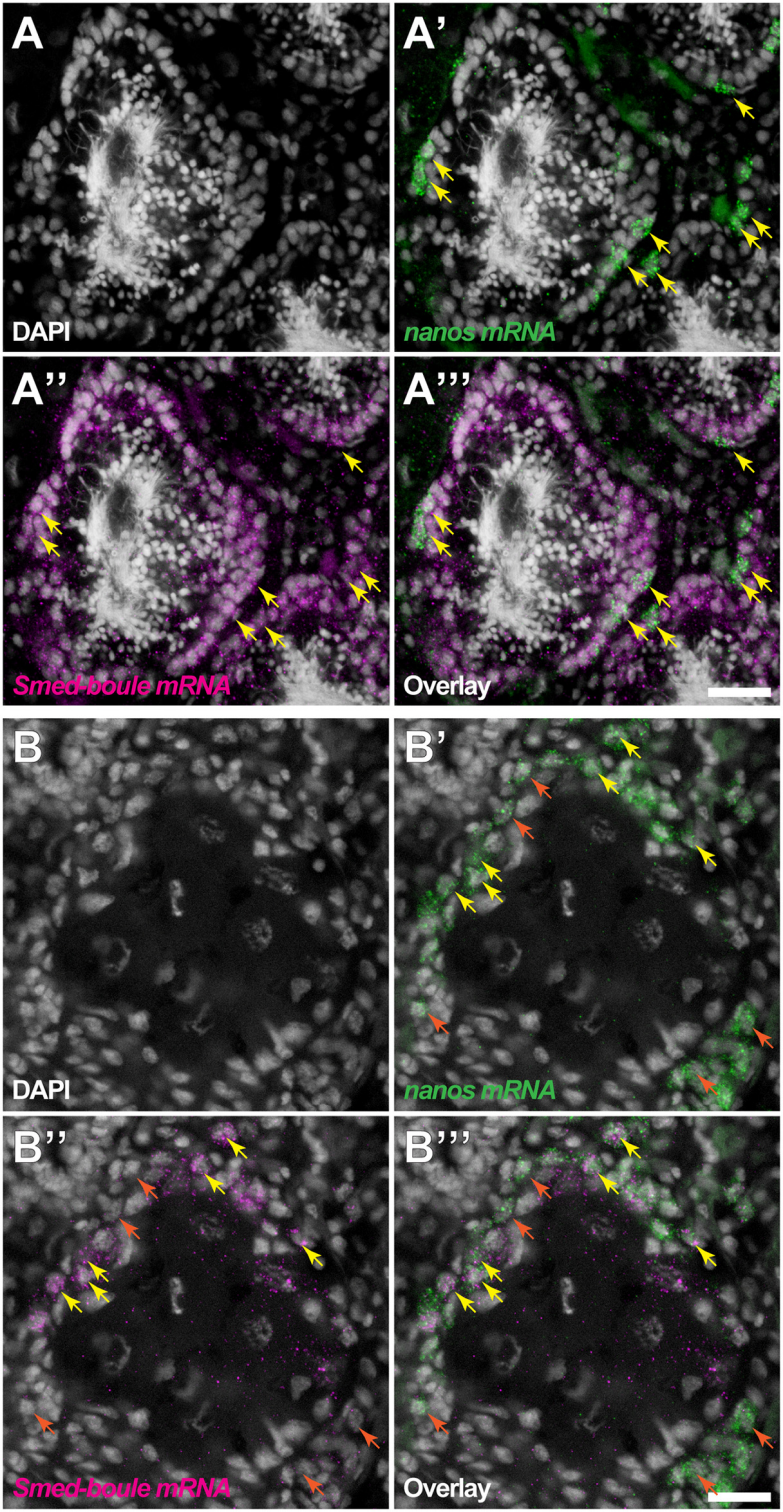
*Smed-boule* is expressed in germline stem cells of testes and ovaries. Detailed analysis by FISH and confocal microscopy of testes **(A)** and ovaries **(B)** of *S*. *mediterranea* reveals *Smed-boule* mRNA (magenta) is detected in germline stem cells, which are recognized through expression of *nanos* (green). *Smed-boule* mRNA was detected in all *nanos(+)* cells of the testes (yellow arrows) **(A”-A”’)**. *Smed-boule* mRNA was also detected in some (yellow arrows) but not all (orange arrows) *nanos(+)* cells of the ovary **(B’-B”’).** DAPI staining **(A and B)** and merged images **(A”’ and B”’)** show the nuclei of all cells present in the imaged frames. Scale bars = 25 μm.

We identified a *boule* homolog in the planarian flatworm *S*. *mediterranea* with a region of amino acid sequence 55% identical with that of the RNA recognition motif of human BOLL (E-value = 1e-23; Fig 1A). The protein encoded by this gene shared highest homology with members of the Boule-like subfamily, as compared with other members of the DAZ family of proteins (Fig 1B). Expression of this gene (from here on referred to as *Smed-boule* or *boule*) was detected by whole-mount *in situ* hybridization (ISH) in testes and ovaries of sexually mature planarians that are actively laying egg capsules (Fig 2A–2D). *Smed-boule* expression was also detected in testis primordia of hatchlings and sexually immature animals (Fig 2D and 2D’). To better understand the distribution of *Smed-boule* expression in testes and ovaries, we performed detailed analysis by double fluorescent *in situ* hybridization (FISH) with the germline stem cell marker *Smed-nanos* [18] (Fig 3). Detection of *Smed-boule* mRNA overlapped with that of *Smed-nanos* in testes (Fig 3A) and partially in ovaries (Fig 3B). The presence of *Smed-boule* mRNA was also robustly detected in the spermatogonial layer of the testes (Fig 3A). Detection of *Smed-boule* expression was not apparent in the soma, and thus we conclude that expression of this gene is restricted to the germline in *S*. *mediterranea*.

**Fig 4:**
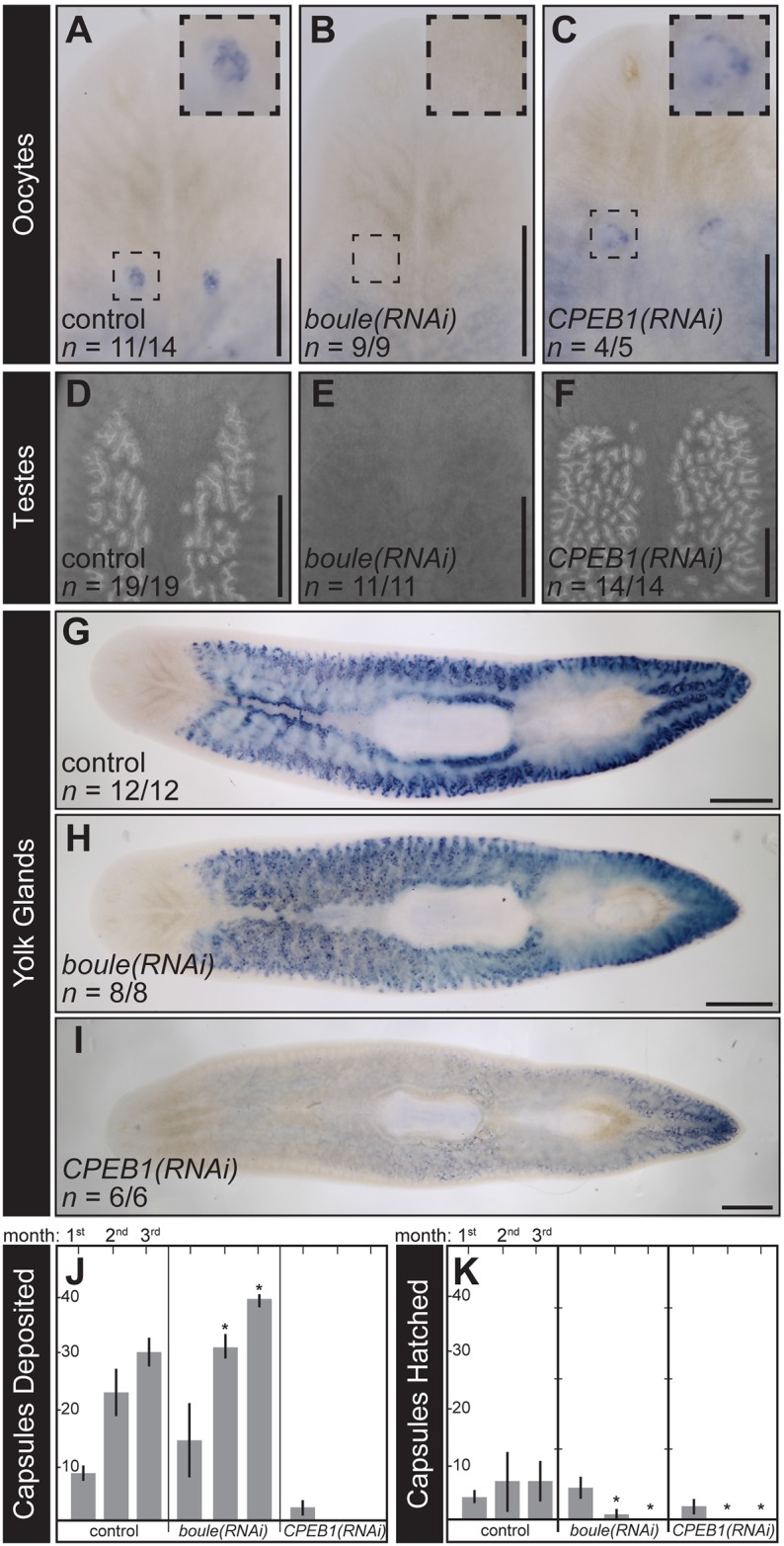
*Smed-boule* is required for germline maintenance and production of fertile capsules. **(A-I)** The reproductive anatomy of RNAi animals monitored in (J-K) was analyzed by whole mount *in situ* hybridization using *synaptotagmin XV* **(A-C)** or *surfactant b* **(G-I)** as oocyte and yolk gland markers, respectively (S1 Fig). DAPI staining was used to visualize sperm development in the testes **(D-F)**. The fraction of animals displaying the phenotype represented by the image is shown at the bottom-left corner of each frame. **(J-K)** Capsule production **(J)** and hatching **(K)** from groups of sexually mature planarians subjected to continuous control, *Smed-boule*, or *CPEB1* RNAi treatments for three months (first, second, and third month represented by column from left to right in each group). Quantification of the number of capsules laid **(J)** and the number of fertile capsules **(K)** show that capsules deposited by *Smed-boule(RNAi)* animals ceased being fertile and that *CPEB1(RNAi)* ceased capsule production as a result of RNAi. Asterisks (*) represent statistically significant results compared to controls of same month by unpaired two-tailed *t*-test (*p > 0*.*05*). Scale bars = 1 mm.

To test the function of *Smed-boule* in planarian germline development and sexual reproduction we subjected planarians to three months of RNAi. Planarians continuously turn over all cells in their body from a continuous population of pluripotent stem cells, which allowed us to assess whether *Smed-boule* is required for normal germline development in sexually mature adults using germ cell markers (Fig 4A–4F; S1 and S2 Figs). Groups of seven sexually mature planarians were fed liver supplemented with 100 ng/μl of double-stranded RNA (dsRNA) twice per week. *Smed-boule* knockdowns (*Smed-boule(RNAi)*) were compared to *control(RNAi)* planarians. DsRNA corresponding to a planarian *Cytoplasmic Polyadenylation Element Binding Protein 1* homolog (*CPEB1)*, which is required for yolk gland development and egg capsule production (below), was administered to an additional group (*CPEB1(RNAi)*) as readout of RNAi effectiveness overtime. At the end of three months of RNAi, we observed that both oocytes (Fig 4B; S2B Fig) and sperm (Fig 4E; S2B” Fig) were absent in *Smed-boule(RNAi)*. No defects in oocyte or sperm development were observed in *control(RNAi)* planarians (Fig 4A and 4D; S2A–S2A” Fig). The testes of *CPEB1(RNAi)* samples were fully developed (Fig 4F), but their ovaries displayed abnormal morphology and distribution of oocyte marker expression (Fig 4C). From these results we concluded that *Smed-boule* is required for development of sperm and ova in *S*. *mediterranea*.

**Fig 5:**
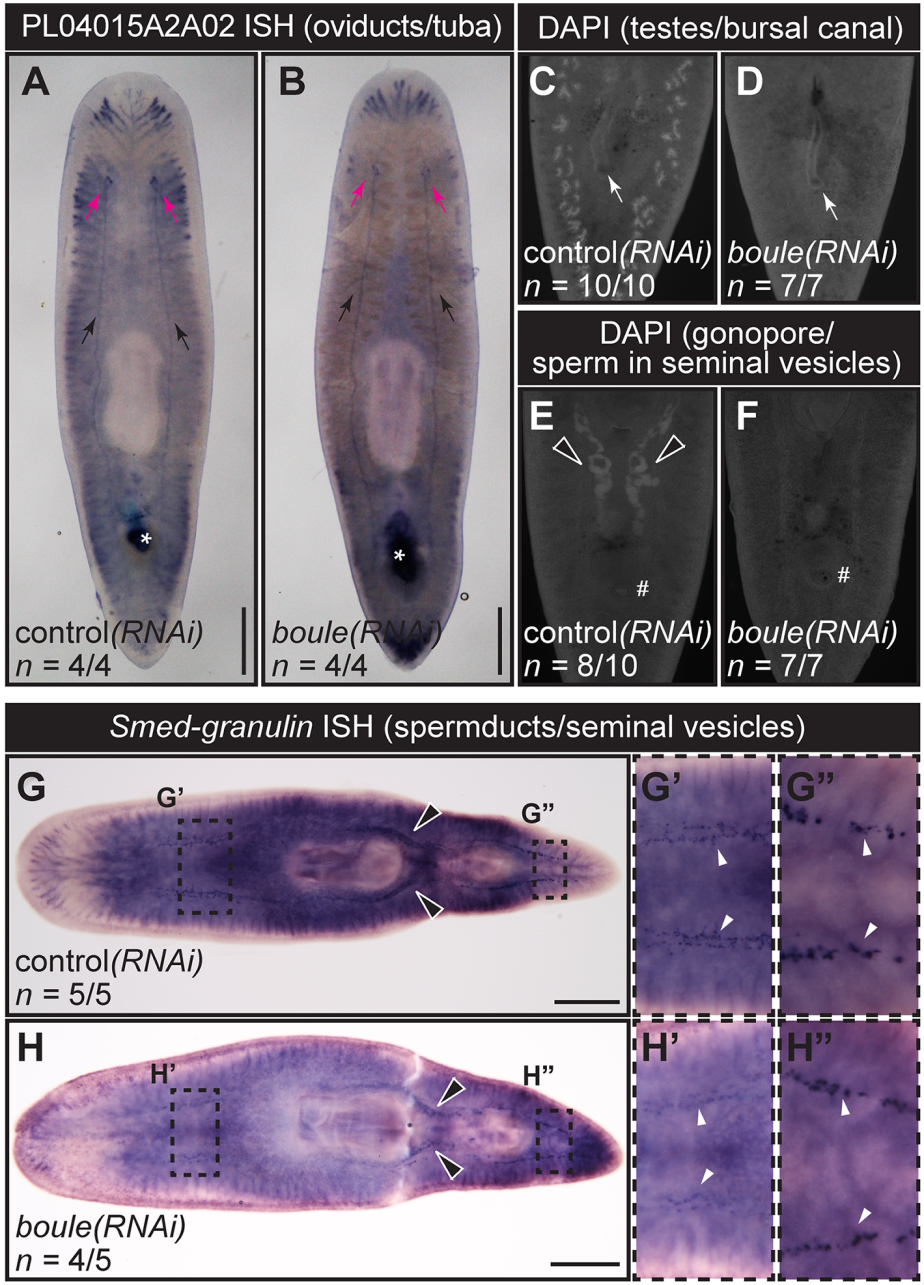
*Smed-boule* RNAi does not affect development of somatic reproductive structures. Whole-mount *in situ* hybridization (ISH) analysis of planarians subjected to three months of control **(A, C, E, and G)** or *Smed-boule* **(B, D, F, H)** RNAi revealed no differences between these two samples in development of oviducts (black arrows in **A-B**), atrium (asterisks in **A**-**B**), tuba (pink arrows in **A-B**), spermducts (white arrow heads in **G’-G”** and **H’-H”**), or seminal vesicle development (black arrowheads in **G-H**). DAPI staining of these samples revealed that development of the bursal canal (white arrow in **C**-**D**) and gonopore (pound sign in **E-F**) were indistinguishable. However, DAPI staining only revealed sperm in testes and seminal vesicles (black arrowheads) of control animals **(E**-**F)**. The fraction of animals displaying the phenotype represented by the image is shown at the bottom-left corner of each frame. Scale bars = 1 mm.

Neoophoran flatworms rely on a particular approach to oviparity in which nutrients for the developing embryo (yolk) are not accumulated in the developing ova. Instead, nutritional support is contributed by yolk cells (vitellocytes), which are transferred from yolk glands to the planarian uterus and encapsulated with embryos during egg capsule deposition. We checked for the presence of yolk glands using the yolk cell marker *Smed-surfactant b* (S1 Fig), which proved to be of comparable abundance and distribution in *control(RNAi)* and *Smed-boule(RNAi)* animals (Fig 4G and 4H). However, the presence of yolk glands in *CPEB1(RNAi)* animals was severely reduced (Fig 4I). We looked for other possible defects in the development of somatic reproductive structures but were unable to find any abnormalities other than the absence of accumulated sperm in the seminal vesicles of *Smed-boule(RNAi)* (Fig 5), which is due to their inability to produce sperm (Fig 4E). There was also a noticeable difference in size of *CPEB1(RNAi)* planarians, which were on average 30.8% larger than control animals maintained under the same conditions (n = 14; unpaired two-tailed *t*-test, *p* < 0.05; S3 Fig). The normality and functionality of the accessory reproductive system in *Smed-boule(RNAi)* was further supported by quantitative analyses of egg capsule production (below).

### Continuous production of sterile egg capsules by *Smed-boule(RNAi)*

As expected, from planarians with underdeveloped yolk glands (Fig 4I), *CPEB1(RNAi)* ceased laying eggs within a month of RNAi (Fig 4J). The rate of egg production in *Smed-boule(RNAi)* was unaffected during the three months of RNAi treatment (Fig 4J). Both the *control(RNAi)* and *Smed-boule(RNAi)* groups continuously laid eggs for the entirety of the experiment (Fig 4J). In fact, an increase of 33% and 30% in egg capsule production was observed in *Smed-boule(RNAi)* when compared to *control(RNAi)* planarians during the second and third months of RNAi treatment, respectively (unpaired two-tailed *t*-test, *p* < 0.05; Fig 4J). Given the surprising result that planarians devoid of gametes continued to deposit egg capsules, we monitored and quantified the number of fertile capsules (yielding progeny) produced by the different knockdown groups for two months after capsule deposition. From this, we discovered that egg capsules produced by animals subjected to two months of *Smed-boule* RNAi completely ceased to hatch (Fig 4K). Egg capsules produced by *control(RNAi)* groups hatched 22% to 48% of the time (Fig 4K). From these results, we concluded that *Smed-boule* function is required for germline development and sexual reproduction in *S*. *mediterranea*, but dispensable for production of egg capsules. Furthermore, the continuous production of egg capsules by groups of *Smed-boule(RNAi)* planarians (Fig 4J) suggested that production and deposition of egg capsules do not require fertilization, contributions from sperm, ovulation, or the presence of oocytes.

### Continuous egg production in planarians regardless of isolation and absence of gametes

Given the fact that *Smed-boule(RNAi)* planarians were capable of producing sterile egg capsules in the absence of germ cells (and therefore fertilization events), we hypothesized that control animals would also produce sterile egg capsules in the absence of fertilization events. To test this hypothesis, we obtained ≤ 1 week-old hatchlings (which lack ovaries, testes, yolk glands, and accessory reproductive organs) and maintained them in isolation for four months under continuous RNAi regimens. Planarians were maintained in isolation throughout the experiment, which allowed us to test whether egg capsule production is independent of signals produced during mating or the presence of potential mates altogether. Since planarians in this experiment were subjected to *Smed-boule* RNAi within a week of being born, which is a point when no sperm has developed, this approach also allowed us to verify that lingering sperm in adult knockdowns used in the previous experiment was not contributing to egg capsule production. Two categories of isolated virgins were maintained on either liver containing *Smed-boule* dsRNA or control dsRNA and were fed twice per week. These animals were expected to grow and eventually reach sexual maturity under these husbandry conditions. The production of egg capsules would only occur if independent from stimuli produced during mating, fertilization, embryonic development and, in the case of *Smed-boule(RNAi)*, the absence of gametes.

**Fig 6:**
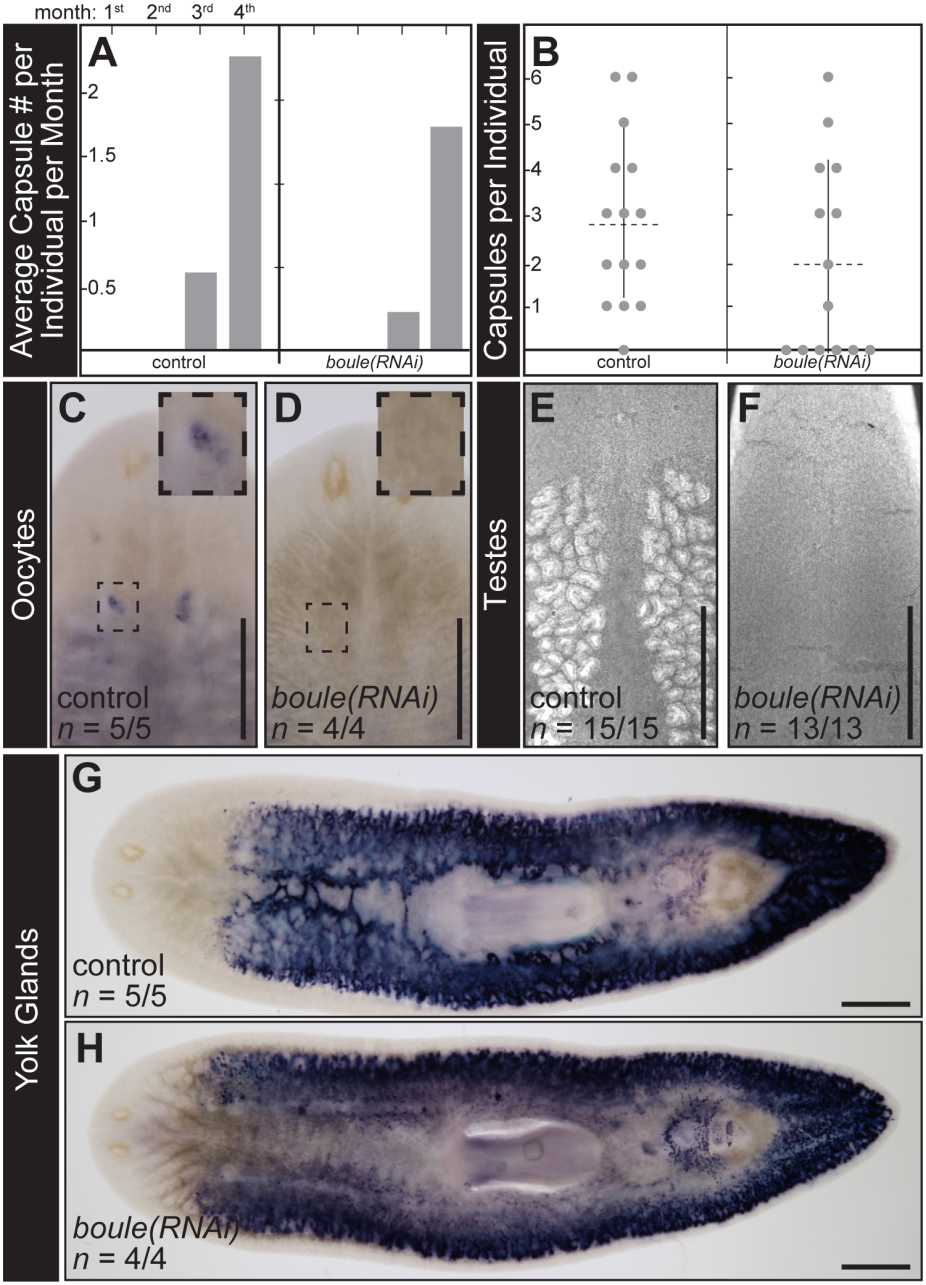
Egg capsules are produced in the absence of gametes or mating events. **(A-B)** Egg capsules produced per month by planarian hatchlings (≤ 1 week old) raised in isolation on a diet of liver containing control (left) and *Smed-boule* (right) dsRNA for four months. The average number of egg capsules deposited per month per isolated individual **(A)**, as well as the total number of capsules deposited per each isolated individual throughout the experiment (single dot in **(B)**) show no significant difference (unpaired two-tailed *t*-test, *p > 0*.*05*) in egg capsule production between control and *Smed-boule(RNAi)* isolated virgins. Dashed lines in **(B)** represent the mean of total number of capsules produced per animal. Vertical lines represent standard deviation **(C-H)** The reproductive anatomy of isolated planarian virgins monitored in (A and B) was analyzed using *synaptotagmin XV* as an oocyte marker **(C-D)**, DAPI staining for testes **(E-F)** and *surfactant b* to assess yolk gland development **(G-H)**. *Smed-boule(RNAi)* lacked oocytes **(D)** and sperm **(F)** seen in control animals **(C and E)**, but developed yolk glands comparably **(G-H)**. Scale bars = 1 mm.

Indeed, both control and *Smed-boule(RNAi)* isolated animals produced egg capsules during the third and fourth months of the experiment (Fig 6A). The number of capsules produced during the length of the experiment by isolated individuals from each category ranged from none to six (Fig 6B). The average number of capsules deposited by individuals in the control category was slightly, but not significantly higher than those of *Smed-boule(RNAi)* (unpaired two-tailed *t*-test, p *=* 0.25) (Fig 6B). As expected from results observed in animals subjected to RNAi in the presence of potential mates (Fig 4J and 4K), none of the egg capsules produced by *Smed-boule(RNAi)* individuals were fertile (n = 0/28 capsules). Capsules produced by control RNAi animals were also completely sterile (n = 0/43 capsules), suggesting that the production of egg capsules in these animals were not due to self-fertilization or parthenogenesis. We verified that normal gamete development was present in control animals at the end of the isolation experiment (Fig 6C and 6E) and absent in *Smed-boule(RNAi)* flatworms (Fig 6D and 6F), which was expected from analyses of knockdowns not maintained in isolation (Fig 4A, 4B, 4D and 4E). We also validated successful development of yolk glands in control and *Smed-boule(RNAi)* planarians raised in isolation (Fig 6G and 6H). Collectively, these results demonstrate that production of egg capsules in *S*. *mediterranea* occurs in response to internal triggers that do not require the presence of a mate, mating, or fertilization events. Furthermore, the production of egg capsules by *Smed-boule(RNAi)* planarians suggests that this trigger is detached from signals originating from sperm and oocyte development or ovulation.

### Spermatogonial stem cells are lost and oogonial stem cells display early oogenesis defects after *Smed-boule* RNAi

**Fig 7:**
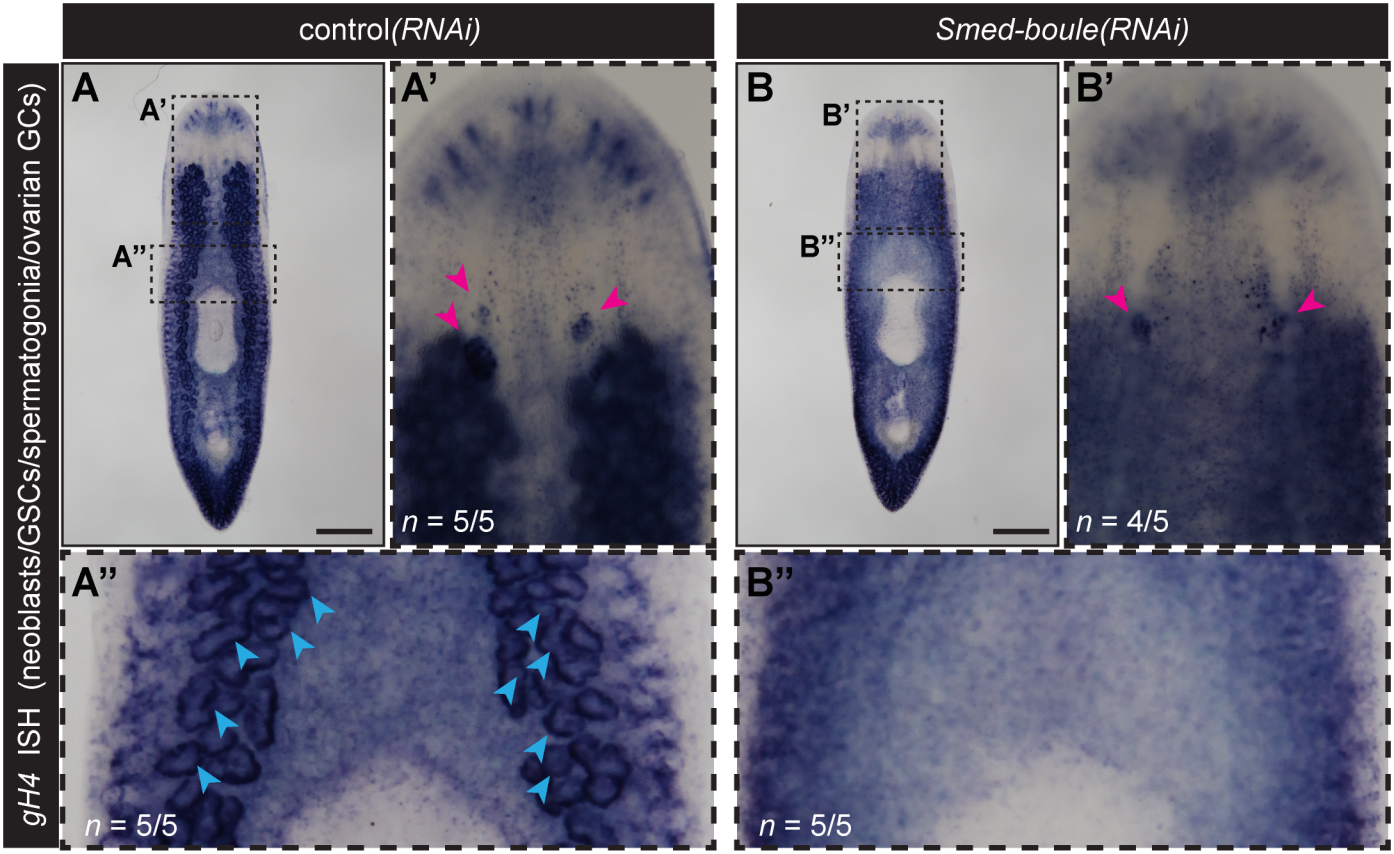
*Smed-boule* is required for maintenance of testicular germ cells, but not for all ovarian germ cells. **(A-B)** Whole-mount *in situ* hybridization (ISH) analysis of neoblast and germ cell (GC) distribution through detection of *germinal histone H4* (*gH4*) mRNA in planarians subjected to three months of control **(A)** or *Smed-boule* **(B)** RNAi. GCs were detected in the ovary region (pink arrowheads) of both controls **(A’)** and *Smed-boule(RNAi)* **(B’)**. Testicular GCs (blue arrowheads) detected in control samples **(A”)** were absent in *Smed-boule(RNAi)* samples **(B”)**. The fraction of animals displaying the phenotype represented by the image is shown at the bottom-left corner of each frame. Scale bars = 1 mm.

**Fig 8:**
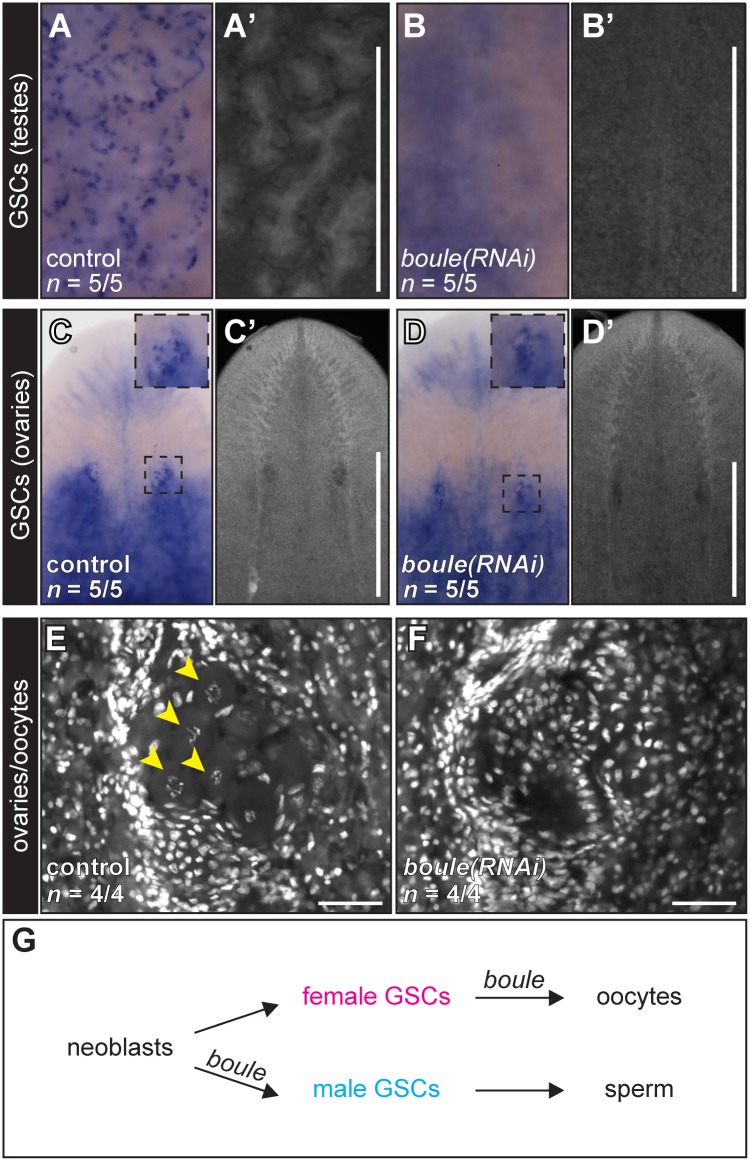
*Smed-boule* is required for maintenance of testicular germline stem cells, but not for ovarian germline stem cells. **(A-D)** Whole-mount *in situ* hybridization analysis of germline stem cell (GSC) distribution visualizing *nanos* expression in the testis **(A-B)** and ovary **(C-D)** regions of planarians subjected to three months of control or *Smed-boule* RNAi. GSCs were specifically absent in the testes region of *Smed-boule* knockdowns **(B)**, but present in the ovary region of both control **(C)** and *Smed-boule* **(D)** knockdowns. Counter-stain with DAPI **(A’-D’)** shows the presence of sperm in testes of control samples (A’), but not *Smed-boule(RNAi)* **(B’).** The fraction of animals displaying the phenotype represented by the image is shown at the bottom-left corner of each frame. **(E-F)** Confocal images of control and *Smed-boule(RNAi)* animals stained with DAPI reveal normal development of oocytes (arrowheads) in control ovaries **(E)** and the absence of oocytes in *Smed-boule(RNAi)* ovaries **(F)**. Scale bars = 1 mm **(A-D)** and 50 μm **(E-F). (G)** Schematic representation of the current model for *Smed-boule* function in differentiation of female GSCs and development and/or maintenance of male GSCs.

We decided to evaluate the severity of germline development defects caused by *Smed-boule* RNAi. The most severe phenotype would be the loss of germline stem cells, which are specified and maintained post-embryonically through neoblast differentiation [18]. Germline stem cells in the planarian ovaries and testes can be identified by the characteristic expression of *germinal histone H4* and *nanos* [18,20,25,26]. We tested for the presence of germline stem cells in *control(RNAi)* and *Smed-boule(RNAi)* by *nanos* ISH after 3–4 months of RNAi (at the end of experiments in Figs 4J–4K and 6A–6B). Whole-mount ISH analysis of *germinal histone H4* and *nanos* expression revealed the presence of germline stem cells in the testes region of *control(RNAi)* animals (Figs 7A and 8A). However, germline stem cells were completely absent from the testes region of *Smed-boule(RNAi)* planarians (Figs 7B and 8B). Surprisingly, germline stem cells in the ovary region of both *control(RNAi)* and *Smed-boule(RNAi)* planarians were readily detectable (Figs 7A’, 7B’, 8C and 8D). Identical results were observed from hatchlings raised subjected to *Smed-boule* RNAi while maintained in isolation (S4 Fig). Furthermore, analysis of germline stem cells in presumptive testis primordia present in asexual strains of *S*. *mediterranea* (Wang et al., 2007) were also lost after *Smed-boule* RNAi (S5 Fig). From these results, we conclude that spermatogenesis defects in *Smed-boule(RNAi)* are due to the absence of male germline stem cells, whereas defects in oogenesis occur further downstream in the differentiation pathway.

To better evaluate the progression of oogenesis in *Smed-boule(RNAi)* ovaries, we analyzed control and *Smed-boule* knockdowns stained with DAPI by confocal microscopy (Fig 8E and 8F; S1 and S2 Movies). DAPI is retained by DNA and allowed for the visualization of numerous large oocytes with condensed chromosomes in the ovaries of control samples (Fig 8E; S1 Movie). In contrast, neither oocytes, cells with condensed chromosomes, or otherwise recognizable mid- to late-oogenic intermediates, were detectable in ovaries of *Smed-boule(RNAi)* flatworms (Fig 8F; S2 Movie). From these results we conclude that *Smed-boule* function is required during the initial stages of oogenesis, sometime before development of primary oocytes, but after specification of ovarian germline stem cells.

The different outcomes observed on germline stem cells of testes and ovaries following *Smed-boule(RNAi)* reveal that these are two fundamentally distinct germline stem cell populations that require *Smed-boule* function at different developmental stages. *Smed-boule* function is necessary for neoblast differentiation into male germline stem cells and/or maintenance of male germline stem cells, whereas ovarian germline stem cells only require *Smed-boule* for progression through early stages of oogenesis (Fig 8G). Furthermore, the severe defects in germline development observed after *Smed-boule* RNAi further support the hypothesis that egg capsule production and deposition occur independently of gametes, ovulation, parthenogenesis, fertilization, mating, or embryonic development in *S*. *mediterranea*.

## Discussion

Collectively, the data presented demonstrate that production and deposition of the egg capsules that ensure development of planarian embryos occur independently of fertilization events. Rather, it seems that egg capsule deposition, at least in *S*. *mediterranea*, is driven by intrinsic signals that are activated once these flatworms grow past a certain size and develop their yolk glands and other accessory reproductive organs. Given these findings, conclusions regarding planarian “fecundity” previously calculated from the rate of capsule production [27,28], may need to be re-evaluated. Additionally, knowledge of the separation between capsule deposition and fertility should assist in the study of planarian germline and embryonic development, as well as in generation of methodologies for transgenesis, which have proven elusive to this point.

Planarian reproduction can occur asexually through transverse fission, or sexually through post-embryonic development of a hermaphroditic reproductive system [7]. In planarians committed to sexualization, the development of gonads and gametes precedes formation of the oviducts, sperm ducts, and copulatory organs [19]. Yolk gland development in *S*. *mediterranea*, which is essential for production of egg capsules, is initiated towards the end of sexual development depending on sufficient nutritional intake and growth. The rate of egg capsule production observed in our experiments (1 to 5 egg capsules per animal per month; Fig 4J) is comparable to those observed in different planarian species both in their natural habitat and raised under laboratory conditions following a similar liver-only diet [29,30]. Therefore, we believe that the conditions used for husbandry of *S*. *mediterranea* in the laboratory are conducive to normal egg capsule production rates, and that this is not the limiting factor in reproductive output. However, the low yield of fertile egg capsules observed from control animals in our experiments (22% to 48%; Fig 4J and 4K) suggests that optimal laboratory husbandry conditions need to established to promote oocyte production, ovulation, or mating (either of which may be rate limiting in actual reproductive output).

How can triggering egg production independently of fertilization be an efficient approach to survival of planarian populations? First, we must consider that in terms of sexual reproduction, *S*. *mediterranea* performs rather poorly under laboratory conditions. This is supported by the studies of Jenkins and Brown [29] who observed *D*. *dorotocephala* yield an average of 16.5 hatchlings per egg (approximately 10-fold higher from what is observed in our laboratory for fertile egg capsules of *S*. *mediterranea*). Studies in *S*. *polychroa* have shown that siblings emerging from a single egg result from different fertilization events, which is possible because sperm from one or more partners can be stored for at least a month after insemination [31]. The ability to store sperm for an extended period of time after insemination, combined with the delay in development of yolk glands in comparison to the rest of the reproductive system, presents a scenario that would benefit from a mechanism that triggers capsule formation independently of copulation. In fact, it would be optimal if the activation of capsule formation also triggers ovulation of the many fully-grown oocytes present in ovaries of sexually mature planarians (Consequential Model; S6A Fig). Since sperm can be stored in the tuba, massive ovulation could maximize the number of hatchlings generated per capsule. Alternatively, encapsulation of multiple embryos in a single egg capsule could also be facilitated by extended storage of zygotes prior to capsule deposition (Complete Autonomy Model; S6B Fig). We are currently unable to differentiate between these two possibilities, or the possibility that passage of oocyte precursors (e.g. oogonial or female germline stem cells) may activate capsule formation. Indeed, oocytes were not detected in *Smed-boule(RNAi)* planarians (*n =* 0/20; Figs 4B and 6D; S2B Fig), but ovaries and oogonial stem cells were readily observed (*n =* 14/15; Figs 7B, 8D and 8F; S4D Fig; S2 Movie). It is possible that the release of early oocyte precursors from the ovary triggers capsule formation. However, this hypothesis is challenged by the fact that dozens of hatchlings often emerge from single capsules of different planarian species, and the observation that the rate of capsule production was not compromised in *Smed-boule(RNAi)* when compared to control planarians (which contained both oocytes and precursors). Nevertheless, current and previous observations do support a model by which a sustainable approach to oviparity could rely on a trigger for capsule formation that is independent of mating, fertilization, or ovulation.

Inside the phylum Platyhelminthes, free-living species (such as *S*. *mediterranea*) are evolutionarily distant from members of parasitic groups (Trematoda, Monogenea, and Cestoda). However, the non-causative relationship between ovulation/fertilization and capsule production appears to be conserved in some cestodes and trematodes, whose dissemination and pathology depend on the continuous production of egg capsules. Parasitic flatworms of the genus *Schistosoma* have been reported to produce egg capsules from females after single-sex infections of mammalian hosts [32,33]. Although female schistosomes depend on interactions with a mate to fully grow and develop their gonads, they are also able to develop some vitelline cells and immature ovaries on their own. Shaw [33] observed the production of infertile capsules from females without male stimuli, probably through mechanisms conserved with those reported here for planarian flatworms. Similarly, parasitic flatworms belonging to the class Cestoda (tapeworms) have been reported to produce unviable egg capsules in the absence of fertilization events when cultured *in vitro* [34,35]. Thus, given that continuous production and deposition of egg capsules is central to dissemination and pathology of different types of parasitic flatworms, the molecular machinery involved in egg capsule production (and not germline development alone) becomes a desirable target for therapeutic developments.

## Materials and Methods

### Planarian culture

A laboratory sexual strain of *Schmidtea mediterranea* [36] was used all experiments, except for those presented with asexual planarians [37] in S5 Fig. Planarian cultures were maintained in 0.75x Montjuïc Salts at 18°C under dark conditions, whereas 1x Montjuïc Salts and 21°C were used for asexuals as per [37]. Planarians were exposed to room temperature and light during weekly feedings of pureed organic beef liver (Vantage USA, Oak Park, Illinois). Experimental animals were starved at least seven days before experimentation.

### cDNA constructs

*Smed-boule* was identified from a collection of *S*. *mediterranea* contig sequences assembled from RNAseq and conventional cDNA expressed sequence tag reads ([38]; https://www.ideals.illinois.edu/handle/2142/28689). A PCR product corresponding to *Smed-boule* ORF sequence was amplified from oligo(dT)-primed total RNA cDNA using 5’-GTTGTTTCAACGGTTCTACTGGCATC -3’ and 5’- GATTATTCCGGACAAAGCTGGACAAG -3’ forward and reverse primers (respectively) and ligated to pJC53.2 [39] after Eam1105I restriction digest. The insert sequence was validated and deposited into NCBI under accession number KT709533.

### *In situ* hybridization and DAPI staining

Fixation and preparation of samples for whole-mount *in situ* hybridization and DAPI staining were performed as per King and Newmark [40]. Colorimetric development for visualization of riboprobes was performed as described by Pearson et al. [41]. *Smed-boule* riboprobes were synthesized using SP6 RNA Polymerase. *Smed-CPEB1* (NCBI accession number KU990884), *Smed-nanos*, were also synthesized from a pJC53.2-based construct [39], whereas *Smed-synaptotagmin XV*, *Smed-granulin* (*grn*), *Smed-surfactant b*, *germinal histone H4*, and a tuba/oviduct marker were synthesized from pBluescript-based clones (PL04017B1F10, PL05005A1F08, PL010001001D12, pBS-*gH4*, and PL04015A2A02, respectively [18,36,42]) using T3 RNA Polymerase. Colorimetric and low magnification analyses of DAPI signals from testes were imaged on a Zeiss Axio Zoom.V16 stereoscope. Confocal images were captured on an Olympus FluoView FV1000 confocal microscope hosted at Wright State University’s Microscopy Core Facility.

### RNAi

Double-stranded RNAi feedings were performed twice every seven days and the protocol was followed as previously described [43]. DsRNA corresponding *Escherichia coli ccdB* sequence, which does not affect planarian development or behavior was used for unaffected control groups. For isolated RNAi samples, each planarian was fed individually and in isolation. For other experiments, planarians were maintained in groups of seven animals.

### Analysis of egg capsule production and hatching

Groups of seven sexual planarians of 0.5 to 0.7 cm size and with a visible gonopore were maintained in glass Petri dishes and subjected to dsRNA feedings as described above. For isolated experiments, single ≤ 1 week-old hatchlings were maintained in isolation in glass Petri dishes throughout the experiment, under the husbandry conditions described above. Isolated planarians were fed liver containing control or *Smed-boule* dsRNA twice per week, at which point any capsules present were collected and isolated. DsRNA corresponding to *E*. *coli ccdB* sequence was used for control samples. Egg capsules were monitored for hatchling events weekly for a period of three months after deposition.

## Supporting Information

S1 Fig Markers used in the analysis of oocytes and yolk gland development. **(A)** Double-fluorescence *in situ* hybridization (ISH) and confocal microscopy detection of *synaptotagmin XV* **(A’)** and *Smed-CPEB1* **(A”)** mRNAs in oocytes of *S*. *mediterranea*. Oocytes are distinguished by DAPI staining **(A)** as large cells with condensed chromosomes in the ovary. **(A”’)** Merged image. **(B)** Whole-mount ISH on sexual strain specimens of *S*. *mediterranea* reveals timing and distribution of *Smed-surfactant b* expression resembling that of yolk glands in the largest animal. Scale bars = 25 μm in **(A)** and 1 mm in **(B)**.
(TIF)

S2 Fig *Smed-boule* is required for oocyte development. *CPEB1* whole-mount *in situ* hybridization (ISH) in sexual planarians subjected control RNAi reveals the presence of oocytes **(A)**, which are absent in *Smed-boule* RNAi planarians **(B)**. Images of DAPI staining in these individuals show the ventral side of control **(A’)** and *Smed-boule(RNAi)* animals **(B’)**, and the presence of testes in the dorsal side of control animals **(A”),** but not in *Smed-boule(RNAi)* **(B”)**. The fraction of animals displaying the phenotype represented by the image is shown at the bottom-left corner of each frame. Scale bars = 1 mm.
(TIF)

S3 Fig A size increase is observed in *CPEB1(RNAi)* planarians. Average size (cm) of sexually mature sized planarians after three months of RNAi treatment reveal a significant enlargement (*) using unpaired two-tailed *t-*test (*p < 0*.*05*) in *CPEB1(RNAi)* animals compared to the size of control or *Smed-boule(RNAi)*.
(TIF)

S4 Fig *Smed-boule* is required for development of testicular germline stem cells, but not for ovarian germline stem cells. **(A-D)** Whole-mount *in situ* hybridization analysis of germline stem cell (GSC) distribution visualizing *nanos* expression in the testes **(A-B)** and ovary **(C-D)** regions of planarian hatchlings raised in isolation and subjected to continuous control or *Smed-boule* RNAi. GSCs were specifically absent in the testes region of *Smed-boule* knockdowns **(B)**, but present in the ovary region of both control **(C)** and *Smed-boule* **(D)** knockdowns. The fraction of animals displaying the phenotype represented by the image is shown at the bottom-left corner of each frame. Scale bars = 1 mm.
(TIF)

S5 Fig *Smed-boule* is required of development of germline stem cells in presumptive testis primordia in asexual strain samples of *S*. *mediterranea*. Detection of germline stem cell (GSC) clusters (white arrows) in presumptive testes primordia of asexual planarians subjected to three weeks of control RNAi (left) or *Smed-boule* (right). Magnified views show detection of GSC clusters in control samples, but not in *Smed-boule(RNAi)*. The fraction of animals displaying the phenotype represented by the image is shown at the bottom-left corner of each frame. Scale bars = 1 mm.
(TIF)

S6 Fig Egg capsules are produced in the absence of gametes or mating events. Models of planarian oviparity that could rely on a shared upstream trigger **(A)**, or on separate and independent pathways **(B)**, for initiating ovulation/fertilization and capsule deposition.
(TIF)

S1 Movie Analysis of oocyte development in *control(RNAi)*. Progression through 3.3 μm confocal sections revealing normal oocyte development in control ovaries stained with DAPI.
(MOV)

S2 Movie Analysis of oocyte development in *Smed-boule(RNAi)*. Progression through 3.3 μm confocal sections revealing the absence of oogenesis in *Smed-boule(RNAi)* ovaries stained with DAPI.
(MOV)
