# Supplementary figures and images for: Germline Defects Caused by Smed-boule RNA-Interference Reveal That Egg Capsule Deposition Occurs Independently of Fertilization, Ovulation, Mating, or the Presence of Gametes in Planarian Flatworms
Source: PLoS Genet. 2016 May 5;12(5):e1006030. doi: 10.1371/journal.pgen.1006030 (PMC4858218; doi:10.1371/journal.pgen.1006030)

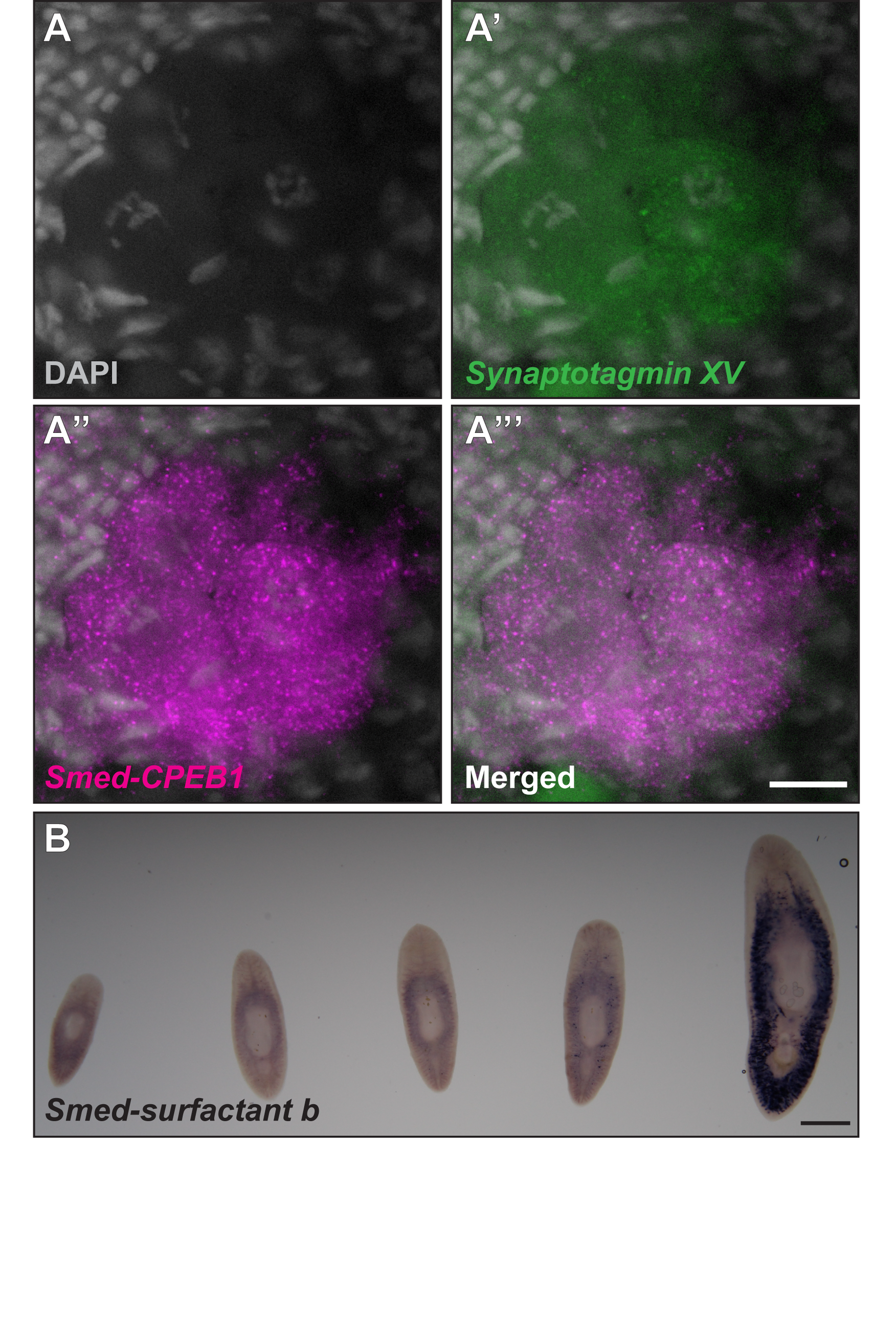

Supplement: S1 Fig — (A) Double-fluorescence in situ hybridization (ISH) and confocal microscopy detection of synaptotagmin XV (A’) and Smed-CPEB1 (A”) mRNAs in oocytes of S. mediterranea. Oocytes are distinguished by DAPI staining (A) as large cells with condensed chromosomes in the ovary. (A”’) Merged image. (B) Whole-mount ISH on sexual strain specimens of S. mediterranea reveals timing and distribution of Smed-surfactant b expression resembling that of yolk glands in the largest animal. Scale bars = 25 μm in (A) and 1 mm in (B). (TIF) [file pgen.1006030.s001.tif]

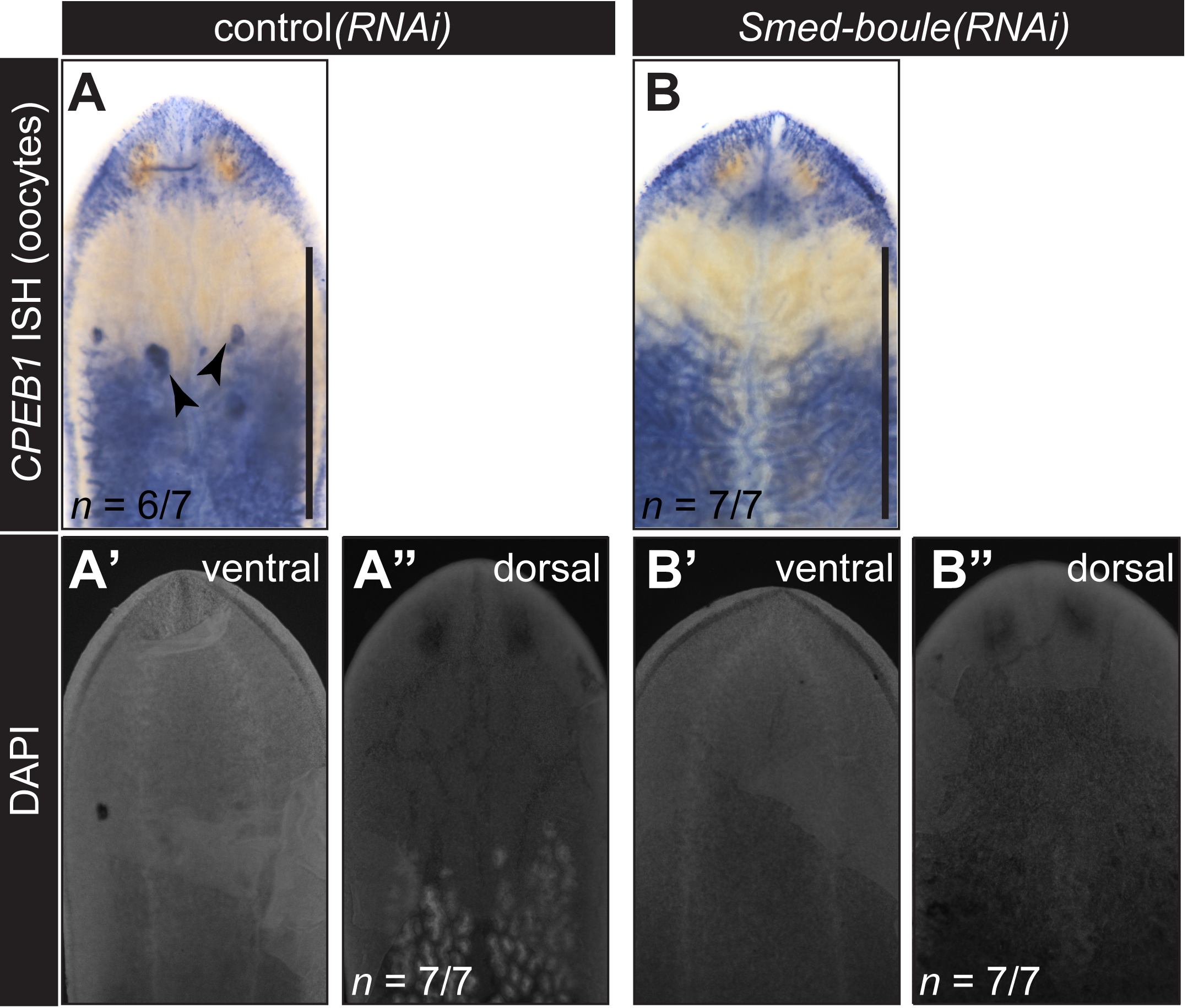

Supplement: S2 Fig — CPEB1 whole-mount in situ hybridization (ISH) in sexual planarians subjected control RNAi reveals the presence of oocytes (A), which are absent in Smed-boule RNAi planarians (B). Images of DAPI staining in these individuals show the ventral side of control (A’) and Smed-boule(RNAi) animals (B’), and the presence of testes in the dorsal side of control animals (A”), but not in Smed-boule(RNAi) (B”). The fraction of animals displaying the phenotype represented by the image is shown at the bottom-left corner of each frame. Scale bars = 1 mm. (TIF) [file pgen.1006030.s002.tif]

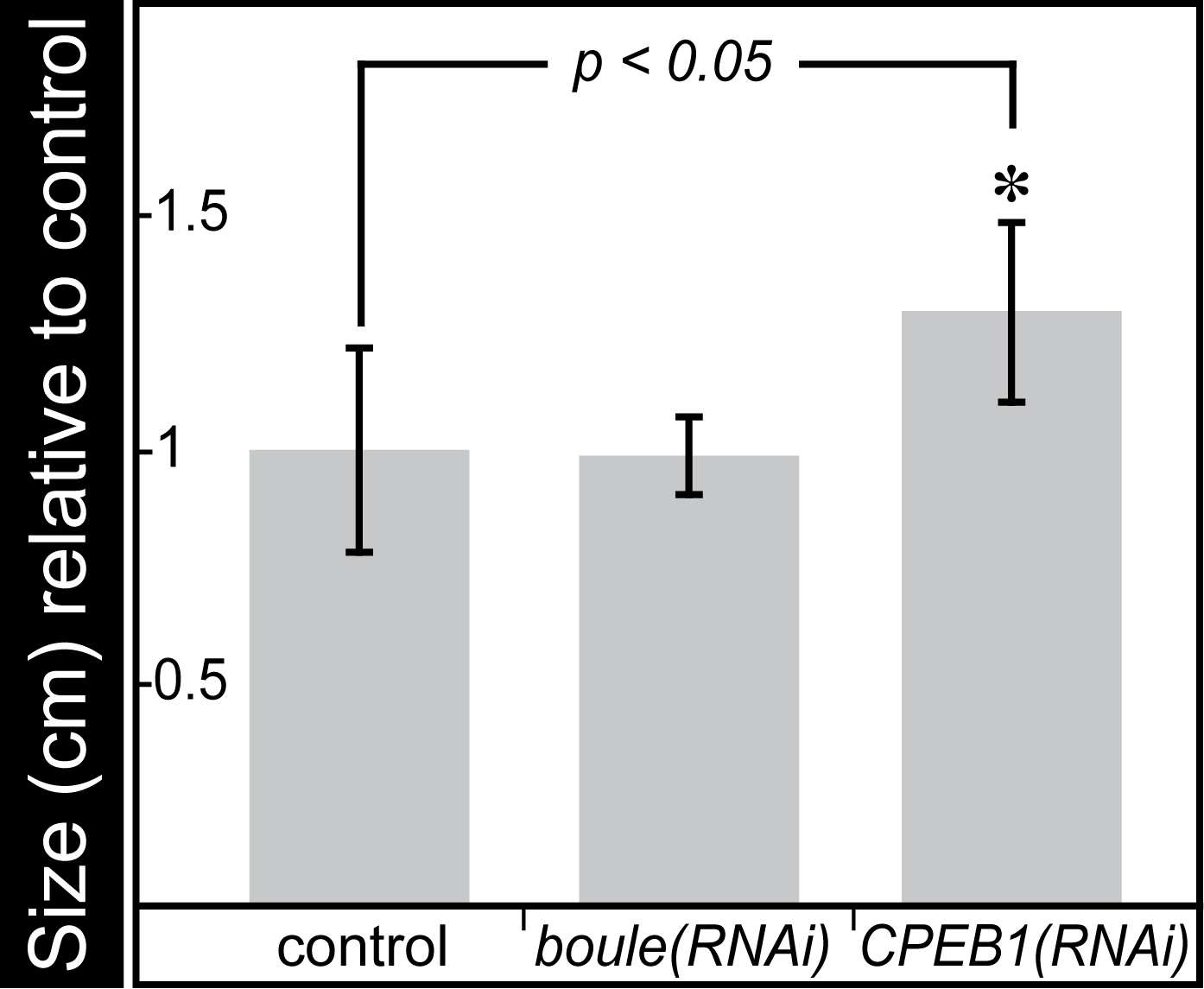

Supplement: S3 Fig — Average size (cm) of sexually mature sized planarians after three months of RNAi treatment reveal a significant enlargement (*) using unpaired two-tailed t-test (p < 0.05) in CPEB1(RNAi) animals compared to the size of control or Smed-boule(RNAi). (TIF) [file pgen.1006030.s003.tif]

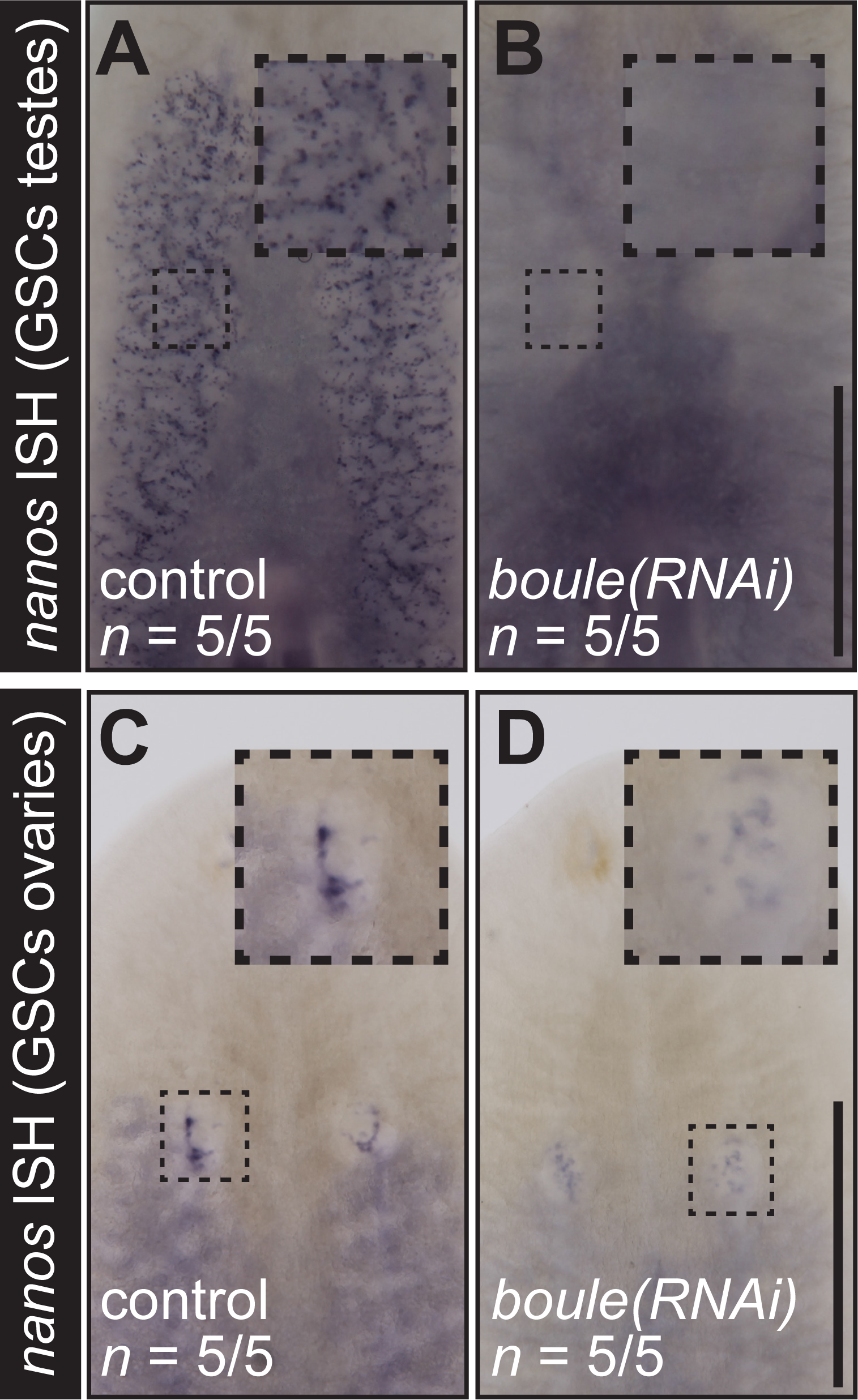

Supplement: S4 Fig — (A-D) Whole-mount in situ hybridization analysis of germline stem cell (GSC) distribution visualizing nanos expression in the testes (A-B) and ovary (C-D) regions of planarian hatchlings raised in isolation and subjected to continuous control or Smed-boule RNAi. GSCs were specifically absent in the testes region of Smed-boule knockdowns (B), but present in the ovary region of both control (C) and Smed-boule (D) knockdowns. The fraction of animals displaying the phenotype represented by the image is shown at the bottom-left corner of each frame. Scale bars = 1 mm. (TIF) [file pgen.1006030.s004.tif]

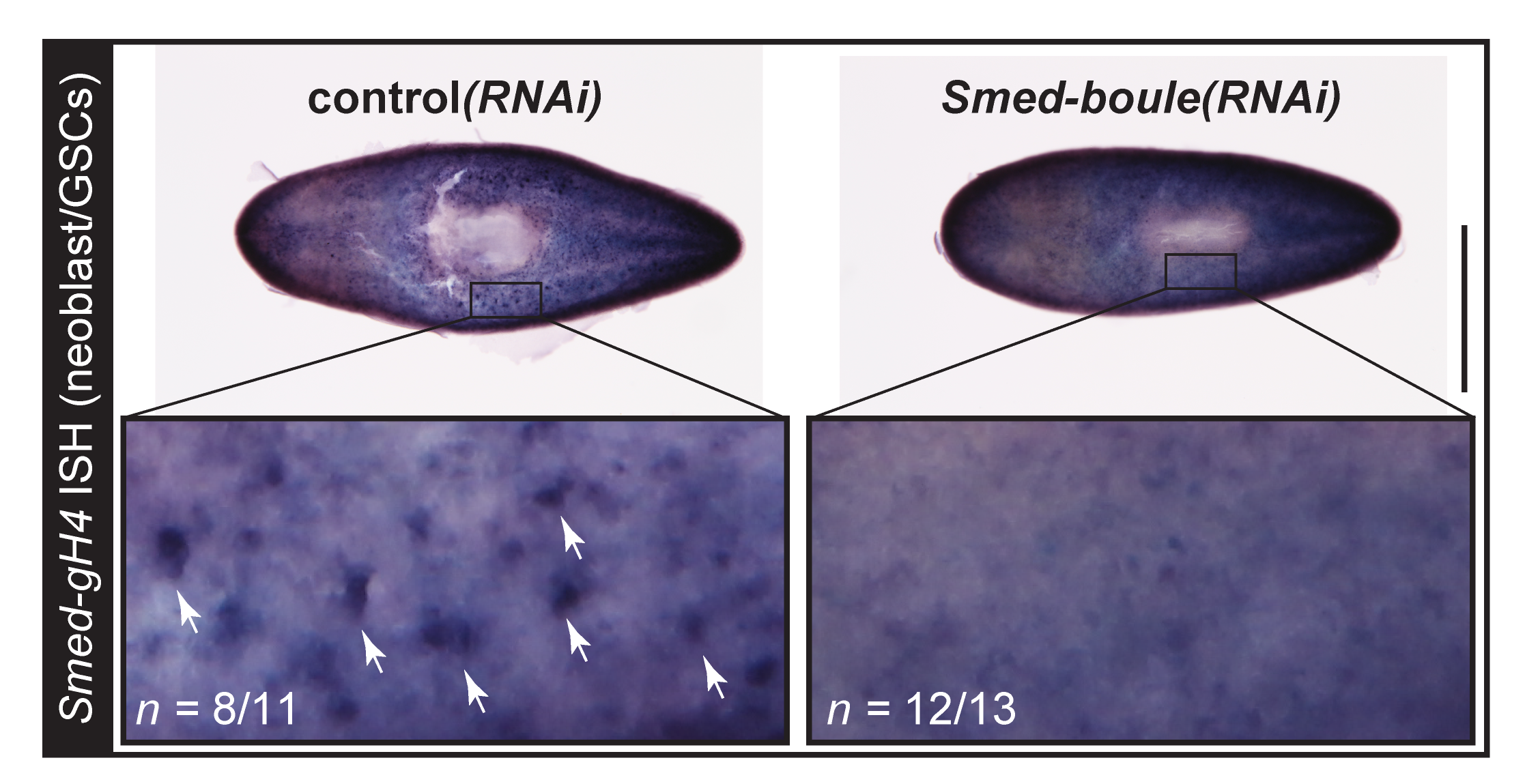

Supplement: S5 Fig — Detection of germline stem cell (GSC) clusters (white arrows) in presumptive testes primordia of asexual planarians subjected to three weeks of control RNAi (left) or Smed-boule (right). Magnified views show detection of GSC clusters in control samples, but not in Smed-boule(RNAi). The fraction of animals displaying the phenotype represented by the image is shown at the bottom-left corner of each frame. Scale bars = 1 mm. (TIF) [file pgen.1006030.s005.tif]

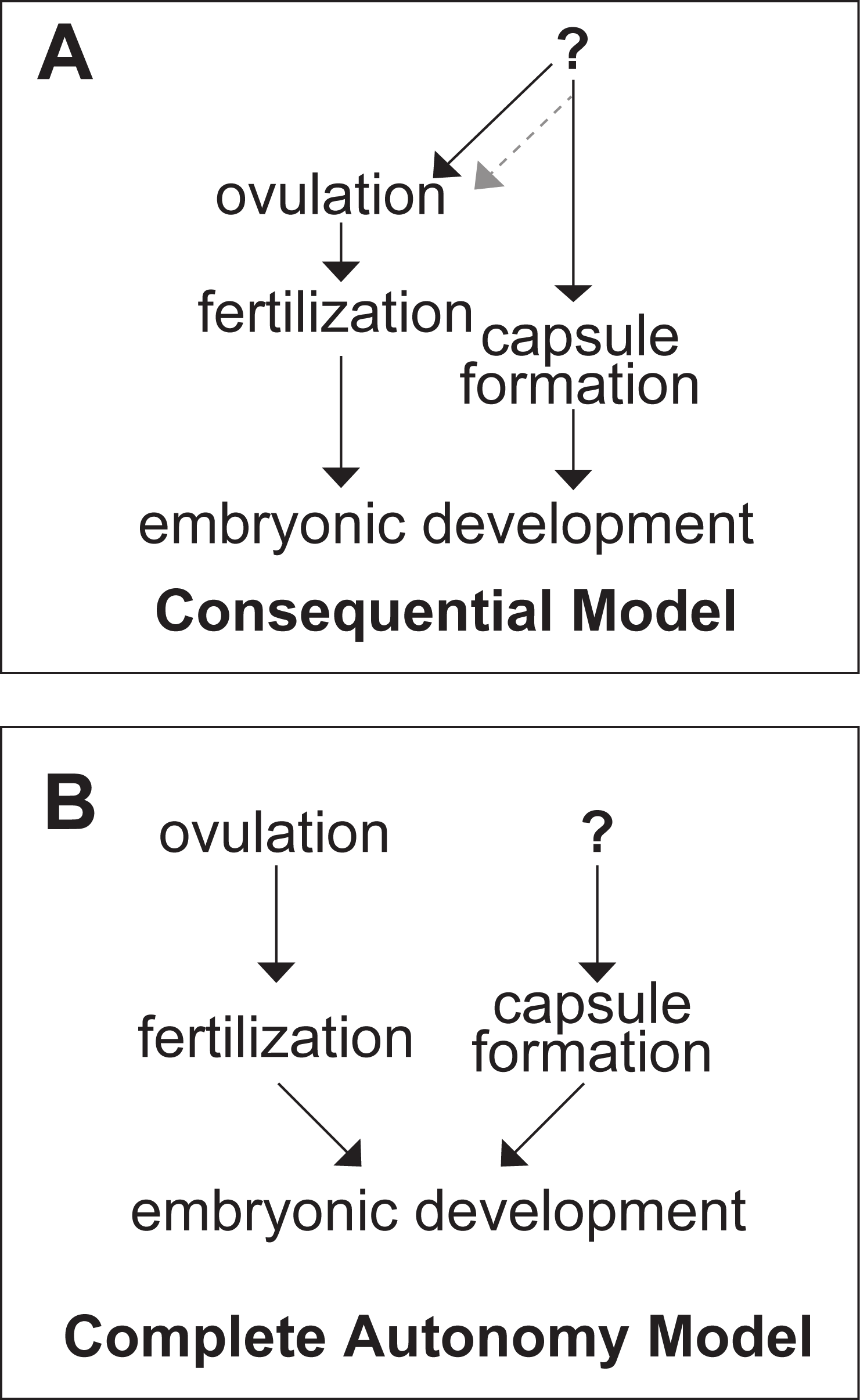

Supplement: S6 Fig — Models of planarian oviparity that could rely on a shared upstream trigger (A), or on separate and independent pathways (B), for initiating ovulation/fertilization and capsule deposition. (TIF) [file pgen.1006030.s006.tif]
